# Direct Patterning of a Carbon Nanotube Thin Layer on a Stretchable Substrate

**DOI:** 10.3390/mi10080530

**Published:** 2019-08-11

**Authors:** Eunji Lee, Hye Jin Kim, Yejin Park, Seungjun Lee, Sae Youn Lee, Taewon Ha, Hyun-Joon Shin, Youngbaek Kim, Jinsik Kim

**Affiliations:** 1 Department of Medical Biotechnology, Dongguk University, Seoul 04620, Korea; 2 Department of Clinical Pharmacology and Therapeutics, College of Medicine, Kyung Hee University, Seoul 02453, Korea; 3 Department of Energy and Materials Engineering, Dongguk University, Seoul 04620, Korea; 4 Center for Nano-Photonics Convergence Technology, Korea Institute of Industrial Technology (KITECH), Gwangju 61012, Korea; 5 Center for Bionics, Korea Institute of Science and Technology (KIST), Seoul 02792, Korea

**Keywords:** Ecoflex, polydimethylsiloxane (PDMS), carbon nanotube (CNT), stretchable sensor, elastomer, oxygen (O_2_) plasma, strain sensor

## Abstract

Solution-based direct patterning on an elastomer substrate with meniscus-dragging deposition (MDD) enables fabrication of very thin carbon nanotube (CNT) layers in the nanometer scale (80–330 nm). To fabricate the CNT pattern with CNT solution, contact angle, electrical variation, mechanical stress, and surface cracks of elastomer substrate were analyzed to identify the optimal conditions of O_2_ treatment (treatment for 30 s with RF power of 50 W in O_2_ atmosphere of 50 sccm) and mixture ratio between Ecoflex and polydimethylsiloxane (PDMS) (Ecoflex:PDMS = 5:1). The type of mask for patterning of the CNT layer was determined through quantitative analysis for sharpness and uniformity of the fabricated CNT pattern. Through these optimization processes, the CNT pattern was produced on the elastomer substrate with selected mask (30 μm thick oriented polypropylene). The thickness of CNT pattern was also controlled to have hundreds nanometer and 500 μm wide rectangular and circular shapes were demonstrated. Furthermore, the change in the current and resistance of the CNT layer according to the applied strain on the elastomer substrate was analyzed. Our results demonstrated the potential of the MDD method for direct CNT patterning with high uniformity and the possibility to fabricate a stretchable sensor.

## 1. Introduction

Over the past few years, the stretchability of a sensor has been considered as one of the significant properties for implementing various technologies such as wearable electronics, artificial muscles, human motion monitoring, and electronic skins [1,2,3]. Owing to this trend, many researchers have attempted to create stretchable sensors with good performance, such as high stretchability and reproducibility. For the high stretchability and reproducibility of a stretchable sensor, excellent deformation properties of the electrodes as well as strong adhesion between the electrodes and elastic polymers are essential [4].

Conventional stretchable sensors have been fabricated by a combination of metal electrodes and elastic polymers such as Ecoflex [5,6], polydimethylsiloxane (PDMS) [6], polyethylene terephthalate (PET) [7], and poly(vinyl alcohol) (PVA) [6,8]. Most electrodes of stretchable sensors have been designed with specific shapes such as wavy, meshed, fractal, or origami and kirigami, resulting in high areal coverage and stretchability in situations requiring rotating, bending, and twisting [9].

Recently, various materials that have high conductivity and better electrical stability for high strains have garnered more attention as alternatives to metal electrodes [10,11]. Conductive polymers, metallic fillers, graphene, and carbon nanotube (CNT) are representative examples of such materials [12]. These materials have a very low thickness and can be coated as a single layer on elastic polymers, resulting in strong adhesion to the polymers. Even the native properties of these materials can be improved by controlling their density, aligning their molecular orientations, and mixing with other materials [13,14]. Among these materials, CNTs are extensively used owing to their structural, electrical, and mechanical properties, such as high aspect ratio, Young’s modulus of 1 TPa, tensile strength of 130 GPa, stretchability up to 25%, and electrical conductivity of 6000 S/cm [15,16,17]. In the CNT-based stretchable sensor, CNTs with a high aspect ratio form an internal conductive network, which outputs the resistance change depending on the external strain. When entangled networks are stretched and reconstructed by an external strain, the electrical resistance of the stretchable sensor changes. Thus, the performance of the CNT-based stretchable sensors depends on the uniformity of the entangled CNT network, which is determined by the fabrication method of the CNT layer, especially by coating and patterning processes. 

The manufacturing process of the CNT-based stretchable sensor can be roughly divided into two processes: Coating and patterning. Coating involves depositing a CNT layer on the elastic polymer and patterning involves forming electrodes with the desired structure [7,16,18]. These two processes can proceed simultaneously or sequentially. Simultaneous coating and patterning the CNT involves spraying the CNT solution on the elastic polymer covered with a sacrificial mask [19]. Although this method allows the easy and cheap fabrication of a stretchable sensor on various substrates including glass, quartz, Indium tin oxide, and polymers, it has limitations in terms of the size and thickness of the CNT pattern. The thickness of the CNT pattern is approximately 20 μm to a few millimeters [20]. A thick CNT pattern has poor adhesion on the substrate and high heterotypic characteristics, resulting in the pattern having poor properties and being easily peeled off [21]. In contrast, the sequential process involves an etching step after the coating of the CNT; the CNT layer is first formed on the substrate via a spin coating method, a dip coating method, chemical or physical vapor deposition, or an epitaxial growth method [22,23]. Then, the coated CNT layer is etched by a photo-lithography process to form the CNT pattern. Although this sequential process can form small, thin, high-quality CNT patterns with strong adhesion properties on the substrate, it is more complex and expensive than the spray coating method. Additionally, growing and etching the CNT requires a large amount of energy, adding a restriction to the substrates that can be selected for use.

Here, we propose a direct patterning method of CNTs with simple meniscus-dragging deposition (MDD) to fabricate a stretchable sensor. MDD is a solution-based deposition method that can form a uniform thin layer of CNTs with a thickness in the range of tens to hundreds of nanometers, resulting in high conductivity and recoverability to strain [24,25,26]. Therefore, manufacturing stretchable sensors using MDD shows great potential; however, a stretchable sensor fabricated using this method is yet to be reported. To this end, we optimized various conditions for the fabrication of a stretchable sensor using MDD. The mechanical and electrical properties of the stretchable sensor substrate, composed of Ecoflex and PDMS, were analyzed according to the proportions of its components. Then, the surface properties of the substrate were determined under various conditions of O_2_ plasma treatment. In addition, the mask for MDD of CNTs was optimized by analyzing the uniformity of the surface and the sharpness of the CNT pattern formed on the elastomer substrate based on five types of masks. After optimization of the substrate, the variation in the thickness of the CNT was analyzed according to the concentration of the CNT solution as well as the CNT deposition times. Finally, various CNT structures were patterned on the elastomer to fabricate a stretchable sensor. The performance of the stretchable sensor was verified by measuring the change in the current and resistance by the change in the strain. Our results show the potential of using MDD as a simple direct patterning method to fabricate CNT-based stretchable sensors.

## 2. Materials and Methods 

### 2.1. Chemicals and Reagents

All chemicals used in the research were of research purity (99.9%) and were used without further purifications. Isopropyl alcohol (IPA) and deionized water (D.W.; 18.2 MΩ·cm at 25 °C) were purchased from iNexus, Inc. (Gyeonggi, Korea). Ecoflex 00-30 series (Ecoflex a and b) were obtained from Smooth-On, Inc. (Easton, PA, USA), and the Siloxane Sylgard 184 silicone elastomer (Polydimethylsiloxane, PDMS-a, PDMS) and its curing agent (PDMS-b) were obtained from Dowhitech Silicone Co., Ltd. (Goyang, Korea). The overhead projector film (OHP) was purchased from Hansol paper Co., Ltd. (Seoul, Korea), and oriented polypropylene (OPP) was obtained from Skyvinyl (Seoul, Korea). Further, polyimide (PI) and Teflon film (25 μm, 50 μm) and Teflon tape (80 μm) were obtained from Alphaflon Co., Ltd. (Seoul, Korea). Ag nanowires were from purchased from Novarials Corp. (Woburn, MA, USA) and Ag flakes (2–3.5 μm, ≤99.9%) from Sigma-Aldrich (St. Louis, MO, USA). Water-dispersed multi-walled CNT (MWCNT; 3 wt%, with purity of > 95%, length: 50 μm) was purchased from US Research Nanomaterial Inc. (Houston, TX, USA).

### 2.2. Preparation of Elastomer Substrates

Elastomers were mixed with Ecoflex and PDMS and tested for three ratio: (1) Ecoflex 100%, (2) Ecoflex:PDMS = 10:1, (3) Ecoflex:PDMS = 5:1. Ecoflex was prepared by mixing the Ecoflex-a (base) and Ecoflex-b (hardener) in a volume ratio of 1:1, and PDMS was obtained by mixing the PDMS-a (base) and PDMS-b (curing agent) in a volume ratio of 10:1. To flatten the elastomer, Ecoflex and PDMS mixture was spin-coated onto the 4 inch Si wafer (iNexus, Inc., Gyeonggi, Korea) at 500, 1500, and 500 rpm for 10, 30, and 10 s, sequentially. The 4 inch Si wafer was covered with Teflon tape which performs as an anti-adhesion layer between Si wafer and elastomer mixture. The mixture substrate was cured at 90 °C for 60 min. 

### 2.3. Surface Masking and Surface Activation with O_2_ Plasma Treatment 

**Figure 1:**
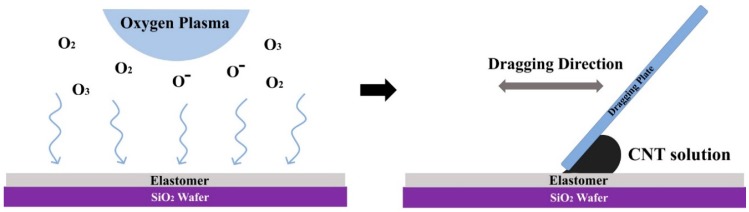
Schematic of direct patterning at the stretchable substrate. O_2_ plasma treatment for direct patterning of a carbon nanotube (CNT) by achieving hydrophilicity (**left**) and CNT deposition by meniscus-dragging deposition (**right**).

An important step in the direct patterning of CNT on the elastomer is to deposit the CNT solution and activate the surface on the desired area. The patterning technique can be achieved by the O_2_ plasma treatment process as shown in Figure 1. The thickness and type of mask material can affect the elastomer surface and resolution of the CNT deposition. Therefore, five types of mask materials were tested in this study, depending on the thickness and type of material: OHP with a thickness of 100 μm (OHP 100), OPP with a thickness of 30 μm (OPP 30), Teflon film with a thickness of 25 μm (Teflon 25) and 50 μm (Teflon 50), and polyimide with a thickness of 25 μm (PI 25). To form the designed pattern on the mask material, a Cricut maker (Cricut, South Jordan) with a Cricut design program was utilized. The patterned masks were loaded onto the elastomer surfaces, and it covered the undesired areas (non-patterning area) of elastomer on the wafer during the O_2_ plasma treatment (RF power: 50 W, oxygen flow rate: 50 sccm, treatment time: 30 s, proceeded with PASCAL-600) to secure the hydrophilicity of surfaces only in areas where the pattern was to be accomplished.

### 2.4. Carbon Nanotube (CNT) Deposition with Meniscus-Dragging Deposition

The elastomer treated by O_2_ plasma was loaded on the MDD system without removing the patterned mask material on its surface. The MDD system is a solution-based deposition system, and it is especially sufficient in depositing very uniform and thin carbon nanomaterials layers over large areas. As shown in Figure 1, after the wafer is loaded on the MDD system, the dragging plate should be placed at an appropriate height (<2 mm) from the wafer. Then, the plate and wafer are fixed such that a contact angle of 45° is achieved. The CNT layer is formed when the plate moves back and forth with the set height and contact angle. To form the CNT layer, diluted MWCNT with D.W., which has the concentration of 1.5 wt%, was used. The CNT solution was injected behind the dragging plate to form a uniform CNT pattern film with a thickness in the nanometer scale by moving the dragging plate. To form the CNT layer, the MDD of ten round trips with three cycles was utilized; we set up the first round trips as dragging the plate in place at once and ten round trips as one cycle. The masking layer should be removed after the deposited CNT layer is dried.

### 2.5. Formation of Electrode and CNT Encapsulation

In order to utilize CNT patterned elastomers as a sensor, CNT patterns were embedded twice inside of an elastomer double layer through a spin coating. In this encapsulation of CNT by the elastomer substrates (i.e., a mixture of Ecoflex and PDMS), external electrodes were connected to the CNT pattern to measure electrical properties with an external measuring equipment. Encapsulation can also prevent external damage of CNT films during measurements. Two methods were utilized to form electrodes with encapsulation. First, an Ag nanowire ink and Ag flake-Ecoflex mixture were selected as materials for the electrode. When the Ag nanowire was used for electrodes, Ag nanowire electrodes were also deposited by MDD at both ends of the CNT pattern with masking OPP film to avoid CNT deposition on unwanted elastomer surface areas. Patterned silver nanowire electrodes secured electrical conductivity through an annealing process at <135 °C within 10 min. Afterward, they were covered with Teflon film to protect them during the packing process with elastomer. To produce the double-layered sensors, an Ecoflex and PDMS mixture was spin-coated onto a wafer at 500 rpm for 10 s, 1500 rpm for 30 s, and 500 rpm for 10 s, followed by a curing stage at 90 °C for 60 min after the annealing process. For electrical measurements, the top layer of the elastomer and the Teflon film covering the electrode were partially removed to create an external exposed electrode for the sensor. 

Afterward, Ag composited with Ecoflex (Ag-Ecoflex) was employed for another electrical layer. The encapsulation process was followed by formation of electrode at once. The Ag-Ecoflex mixture was composited with Ag flake dispersion in IPA and Ecoflex. The mixture of Ag-Ecoflex was spin-coated onto the patterned CNT layer at 500 rpm for 10 s, 1500 rpm for 30 s, and then cured at 90 °C for 60 min. All the processes for the formation of the encapsulated CNT pattern connected with electrodes in elastomer substrate are shown in Figure S1 of supplementary material. 

### 2.6. Contact Angle and Cross Points Measurement

Elastomer treated with O_2_ plasma for 0–90 s, was placed on the measurement table of a contact angle analyzer (Phoenix 150 and Surfaceware 8, SEO, Suwon, South Korea) to measure the contact angle between a water drop and the elastomer surface. Since the hydrophilicity conferred by O_2_ plasma decreases over time, contact angle measurements were made within 15 min after treatment. The contact angle was measured by the contact angle analyzer 15 min after, and 30 min after the plasma treatment. These contact angles were then compared. Optical images of the contact angles were obtained by the contact angle analyzer.

Cross points were counted to present quantitate values for crack occurrence, which can be defined as the intersection of the crack line in the surface found after O_2_ plasma treatment, as shown in the supplementary files (Figure S2). To measure cross points of elastomer surface after O_2_ plasma treatment, the intersection points of crack lines were counted in optical images of the elastomer surface, which were treated for 0–90 s. The optical image was taken to have 8 mm wide and 4.5 mm high image plane.

### 2.7. Electrical Properties Measurement

Electrical properties and resistance of the CNT pattern were measured by a probe station and source meter (2401, Keithley Instruments, Solon, OH, USA). After probe tips were contacted to each end of CNT pattern, currents were measured while voltages were supplied by the source meter. 

### 2.8. Mechanical Properties Measurement

Mechanical properties were measured with a digital strain gauge (DS2-2N, ZLINK3E, IMADA Co., Ltd., Toyohashi, Japan). To stretch the elastomer samples or sensors, a fixed strain gauge and motorized stage (Smart Actuator, SAN6510-200S+3SL, i-ROBO, Gwangmyeong, Korea) were used. Samples were fixed to one side of the strain gauge and another to the motorized stage. The motorized stage was controlled to move back and forth for a certain length to deform the elastomer substrate. Most of the experiments in this study were preceded by loading 100% strain on tested samples, which is determined by the length of samples between the strain gauge and the stage.

### 2.9. Image Processing for Analysis of Uniformity and Sharpness 

Uniformity and sharpness of the CNT pattern were analyzed by computational processing of the optical images of patterns via the Image Pro 10 software (Media Cybernetics Inc., Washington, DC, USA). The quality of the CNT pattern varied according to the type of masking material, which could be defined by sharpness and uniformity. Many types of research in which it is important to define the pattern’s uniformity and sharpness, such as panel displays, perform these analyses [27,28]. The uniformity of the CNT pattern can be defined as follows (1);

(1) $$U\;\left(\%\right)\;=\left(\frac{I_{LI}}{I_{avg}}\right)\times 100,$$

where U represents the uniformity of the CNT pattern, $I_{LI}$ is the average of the lowest ten intensity values of the CNT pattern, and $I_{avg}$ represents the average intensity values of the CNT pattern in the line profile. In CNT line profile analysis, the intensity is determined according to the intensity level of pixels in line profile of the optical image. Generally, the area where the CNT is deposited has a low intensity. Therefore, uniformity was obtained by calculating the percent ratio between the lowest intensities and the averaged intensities of a dark area, which were considered as CNT patterns.

Sharpness can be defined by comparing the line profile in the achieved CNT pattern images as follows (2);

(2) $$S\;\left(\%\right)=\;\frac{\left(W_{max\;}\;-\;W_{min}\right)}{\left(W_{max}\;+\;W_{min}\right)}\times 100,$$

where *S* indicates the sharpness of the CNT pattern’s edge, $\;W_{max}$ is the maximum width of the dark area in the CNT pattern line profile, and $W_{min}$ is the minimum width of dark area. Generally, patterns have a blurred edge when the patterns are magnified. Briefly, the $\;W_{max}$ is the counted number of pixels between the outside of blurred edges that are right next to the bright area, the non-patterned area, which has high intensity of pixels. The $W_{min}$ is the counted number of pixels between inside of the blurred edges that are obviously considered as the CNT pattern. Consequently, the sharpness can be accomplished with comparison between counted pixels, which is considered as the minimum width and maximum width of the CNT pattern. The sharpen pattern has almost the same value of minimum width and maximum width of the CNT pattern and lower *S* (%); the sharpen pattern has less blurredness.

## 3. Results and Discussion

### 3.1. O_2_ Treatment of the Stretchable Substrate Surface

**Figure 2:**
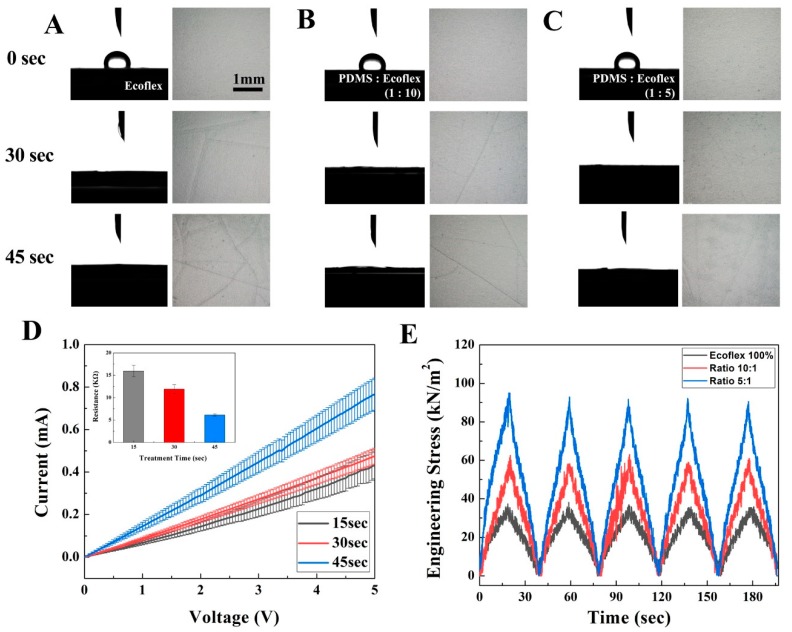
Electrical and mechanical properties of mixture of Ecoflex and polydimethylsiloxane (PDMS) varied by O_2_ plasma treatment time. (**A**)–(**C**) Optical images of contact angle (left) and surface crack of elastomer (right). The substrate mixture ratio is (**A**) Ecoflex:PDMS = 100:0, (**B**) Ecoflex:PDMS = 10:1, and (**C**) Ecoflex:PDMS = 5:1. (**D**) The current–voltage curve of deposited CNT on the substrate (Ecoflex:PDMS = 5:1), (**E**) by stretching the double-layered substrate to 100% strain, the mechanical stress of three different types of substrate changed in kN/m².

The O_2_ plasma treatment was demonstrated to accomplish the hydrophilicity of the elastomer’s substrate. This hydrophilicity is a very important variable to deposit CNT layer on an elastomer substrate. As shown in Figure 2, the contact angle was measured on the O_2_ plasma-treated elastomer at different treatment times to confirm hydrophilicity. After the O_2_ plasma treatment, the current of the deposited CNT layer according to the applied voltage was also measured to predict the uniform formation of a CNT layer followed by the plasma treatment.

The elastomer can be changed to have different mechanical characteristics after the plasma treatment. Since cracks were observed depending on the oxygen dose and pressure for sample activation, we measured the cracks on elastomer’s surface and mechanical strength of the elastomer [29]. The mechanical strength of the elastomer was measured by a digital push/pull gauge mentioned in 2.8. To measure the strength of the elastomer, non-CNT deposited elastomers were prepared, and elastomers were cut to a 2 cm width and 1 cm height. Tensile strain was applied for 30 s with five repetitions. Tensile strength was obtained when substrates were stretched to 100% strain. 

As shown in Figure 2A–C, the hydrophilicity and interfacial attack by O_2_ plasma treatment were measured. Figure 2A–C, respectively, show the results for the elastomer substrate, which have a mixture ratio of Ecoflex and PDMS 100:0, 10:1, and 5:1. Quantitative measured data for Figure 2A–C were shown in Table 1. Through O_2_ plasma treatment, all the substrates have a tendency to be hydrophilic. As shown in Table 1, values of approximately 102.32 ± 3.75°, 102.73 ± 1.29°, and 96.51 ± 2.36° were respectively measured before O_2_ plasma treatment for the elastomer substrate, which have a mixture ratio of Ecoflex and PDMS 100:0, 10:1, and 5:1. All the substrates have 9–12 degree contact angles after the O_2_ plasma treatment; detailed values are shown in Table 1 according to the conditions. The values were sufficient to determine that the substrate had hydrophilicity. More contact angles data for Ecoflex and PDMS 100:0, 10:1, and 5:1 are shown in Figure S3, Tables S1 and S2 of the supplementary material; treatment time of 0, 15, 30, 45, 60, and 90 s. 

However, the substrate had an interfacial attack through the O_2_ plasma treatment. The bright field images of Figure 2A–C (images on right line at each Figure 2A–C) are the top view of substrates to show the cracks on substrate. Before the O_2_ plasma treatment, the substrates had no cracks; each image for 0 s of Figure 2A–C. After the O_2_ plasma treatment for 30 s, the cracks occurred on the elastomer substrates. There were no cracks on the substrate treated by the plasma treatment during 15 s. However, obviously more cracks existed when treatment time was 45 s than 0 s or 30 s treatment time at all three kinds of elastomer substrate. Much more cracks were accomplished due to more O_2_ plasma treatment time. Until a treatment time of 15 s, there were no cracks on the elastomer substrate.

For a quantitative analysis of crack’s occurrence due to O_2_ plasma treatment, cross point among the crack was utilized. The cross point can be accomplished when numerous line profiled cracks exist and cross among them as mentioned in 2.6. The cross point was counted and considered as the number of cracks on the surface by O_2_ plasma treatment. As summarized in Table 1, the number of cross points was introduced according to the treatment time for each kind of elastomer substrate. Similar to the tendency of cracks, which were considered with the cracks images of Figure 2A–C, the cross points were also increased according to the treatment time. The number of the cross points, which counted by the analysis of cross points, was 0 on the all substrate treated by O_2_ plasma during 15 s. However, the number of the point was 4.8, 2.7, and 0.8 on the substrates with a ratio of the Ecoflex and PDMS of 100:0, 10:1, and 5:1, respectively, in the O_2_ plasma treatment time of 15 s. These results indicated that two conditions were sufficient to form hydrophilicity and less cracks on surface. More data of crack for Ecoflex and PDMS 100:0, 10:1, and 5:1 were shown in Table S3 of supplementary material; treatment time of 0, 15, 30, 45, 60, and 90 s. Figure S3 in the supplementary material shows the example of a cross points analysis of 45 s treated elastomeric substrate; red dots were measured points, which were derived by the cross of line patterned cracks.

For more specific optimization of the treatment time, variation of the current on the deposited CNT on three types of elastomer substrates was analyzed. The variation was calculated through the current–voltage curve as shown in Figure 2D, and elastomer substrates were treated by O_2_ plasma for 15 s, 30 s, and 45 s, respectively, before CNT deposition. The measured values of resistance were approximately 15.95 ± 1.32 kΩ, 11.92 ± 0.98 kΩ, and 6.12 ± 0.24 kΩ for the substrate treated by O_2_ plasma for 15, 30, and 45 s, respectively. 

The value of variation measured at the CNT on the substrate treated by O_2_ plasma for 30 s was lower than the value on the substrate treated for 15 s. The results indicated that the CNT layer was more uniform when deposited on the substrate treated by O_2_ plasma for 30 s than 15 s. Although the CNT layer on the substrate treated by O_2_ plasma for 45 s showed less variation than in other conditions (±0.24 kΩ), this treatment time was already excluded via the cross points analysis, so, we focused on treatment time as 30 s.

For elastomer substrates with a mixture ratio of Ecoflex and PDMS 100:0 and 10:1, there were also many cracks at 30 s of treatment time. The lowest number of cross points was acquired from substrates with a mixture ratio of Ecoflex and PDMS 5:1; 0.8 cross points was measured at 30 s of treatment time.

To obtain the hydrophilicity of the surface, the surface was treated by O_2_ plasma for 30 s. After 15 min, which was the time required for post processing such as loading substrate to MDD system, dropping the CNT and so on, the hydrophilicity of the substrate was confirmed by comparing the contact angle (Table S2). From the results, it was demonstrated that the hydrophilicity of the surface sufficiently remained for 15 min.

In order to confirm the mechanical characteristics of the substrate depending on the cracks on the surface, the substrate, which was treated by the O_2_ plasma for 30 s, was covered with the same materials of the substrate without patterning the CNT layer. Elastomer substrates with Ecoflex and PDMS ratios of 100:0, 10:1, and 5:1 were utilized. After 30 s of O_2_ plasma treatment on a single layer of elastomer substrate, another elastomer layer was formed via spin coating and baking. Briefly, this elastomer with a double layer also has the properties shown in Figure S1 of the supplementary material: No “surface masking”, “CNT deposition”, and “Mask remove and CNT dry”. As shown in Figure 2E, mechanical stress due to the stretching force was measured for all three kinds of elastomer substrates. Tensile stress was applied five times for 20 s with 20 s of recovery time; the total measuring time was 200 s. Engineering stress of elastomers were calculated by dividing the force by the initial cross section area; Ecoflex and PDMS 100:0 were measured as 173.4 ± 1.9 μm, 10:1 as 164.1 ± 1.2 μm, and 5:1 as 163.6 ± 1.8 μm, which are summarized in Supplementary Figure S4 and Table S4. Elastomer substrates, which are Ecoflex and PDMS 100:0, 10:1, and 5:1, have a stress as approximately 34.9 kN/m², 61.2 kN/m², and 85.5 kN/m², respectively. The curve was continuously measured and had a sharp peak without any breaking points, which were considered the breaking or fragile of the double elastomer layer.

Although some small cracks were observed on the surface of the elastomer substrate, which were treated by O_2_ plasma for 30 s, the treatment time was optimized to 30 s because of its low current variation and good adhesion with another elastomer layer.

### 3.2. Mask Selection

For direct patterning of CNT on the elastomer substrate, the mask selection was very important. The mask acts as a sacrificial layer in CNT deposition by MDD to obtain a specific pattern and determined the areas for pattern and non-pattern. Selection of the mask was accomplished with five kinds of mask to optimize which kind of mask is sufficient to demonstrate direct patterning on elastomer substrate. The information regarding material and thickness of utilized masks are shown in Table 2.

**Figure 3:**
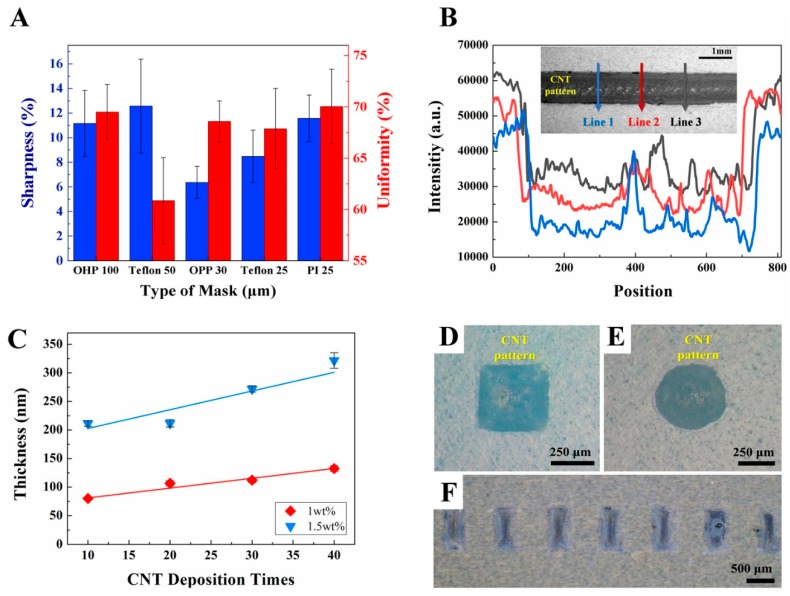
Optical properties and thickness of CNT films deposited on elastomer substrate by meniscus-dragging deposition (MDD) for mask selection and showing the thickness and types of patterns, which can be formed by direct patterning. (**A**) The average values and standard deviation of sharpness (blue, %) and uniformity (red, %) of CNT patterns are varied by masking materials. (**B**) Optical image of CNT with marked lines and intensity of line profiles when the “OPP 30” mask was used. (**C**) Thickness of the 1 wt% CNT layer (red) and 1.5 wt% CNT layer (blue) based on the number of CNT depositions and the results of atomic force microscopy (AFM) analysis. Optical images of CNT in (**D**) a square pattern, (**E**) a circle pattern, and (**F**) square arrays with “OPP 30” mask.

Uniformity and sharpness were defined for the quantitative analysis as shown in Equations (1) and (2) for quantitative analysis of acquired patterns for each mask. “Uniformity” refers to how the pattern can be fulfilled with CNT, “sharpness” refers to how clear patterns can be accomplished. These two factors are important to obtain good patterns. The uniformity and sharpness depending on the types of mask were tested and the results are plotted as shown in Figure 3A. The analysis of uniformity and sharpness in Figure 3A was progressed with analysis of pixel intensity according to the position (pixels) as Figure 3B. Inset image of Figure 3B was CNT deposited line pattern with “OPP 30” as an example. All analyses for other masks were also accomplished by these analyses of the CNT pattern image. The pixel intensity was low from position 100 to 700, which can be considered as the deposited CNT area. The intensity decreased or increased around position 100 and 700, considered as the edge of the CNT pattern. Some higher pixel intensities were acquired from positions 400 to 500. The quantitative values of intensity of the pixel were measured. The graph of Figure 3A was acquired from the measured intensity of pixel with a calculation by Equations (1) and (2). The calculation was derived as follow the marked lines from the top to bottom direction of images for the selection of the mask.

The uniformity of the CNT pattern ranged from approximately 60.86% ± 8.42% in “Teflon 50” to 70.04% ± 7.27% in “OHP 100”, as shown in Figure 3A. The mean values of uniformity of all the masks except “Teflon 50” were 70%; the values for “OHP 100”, “OPP 30”, “Teflon 25”, and “PI 25” were not very different. When “OPP 30” was used, the variation of uniformity was its lowest, at ±4.02%. The deposited pattern, clearly shown in the inset of Figure 3B, had a largely dark area as designed and some bright sections in the middle of the CNT. This phenomenon is a necessary occurrence in solution-based patterning and is the reason why we tried to analyze uniformity according to the type of mask. Furthermore, the sharpness of the CNT pattern ranged from approximately 6.37% ± 2.60% in “OPP 30” to 12.57% ± 7.62% in “Teflon 50” as shown in Figure 3A. Low values of sharpness could be considered given that a greater degree of sharpening could be accomplished given the definition of sharpness. In the range of the acquired value of sharpness, “OPP 30” had the lowest value of sharpness as 6.37% ± 2.60%.

The results of these analyses showed that approximately 70% uniformity was achieved for the CNT pattern at “OHP 100”, “OPP 30”, “Teflon 25”, and “PI 25,” and the lowest value of sharpness of the CNT pattern was achieved with “OPP 30”. Furthermore, “OPP 30” also had the lowest variation in uniformity. Although the comparison among five kinds of masks was relative, “OPP 30” could be considered sufficient for sharpening the patterning of a CNT on an elastomer and making them uniform. In the rest of our experiments, “OPP 30” was used to form CNT patterns.

### 3.3. CNT Pattern with Direct Patterning

As shown in Figure 3C, the CNT layer formed was several tens to hundreds of nanometers thick. Several circles or rectangular shapes, tens to hundreds of micrometers wide, were seen on elastomer substrate with direct patterning. This was accomplished after the substrate was modified with a O_2_ plasma treatment and the selected mask.

An atomic force microscope (AFM, Park Systems, Suwon, Korea) was utilized to confirm the thickness. Two kinds of solution containing CNT, at concentrations of 1 and 1.5 wt% each, were also utilized in this experiment. The thicknesses were measured based on the CNT deposition times, which was the amount of MDD repetitions. As shown in Figure 3C, the thickness increased with deposition. For the 1 wt% CNT, controllable thicknesses of approximately 80 nm to 130 nm were achieved, and for the 1.5 wt% CNT controllable thicknesses of approximately 210 nm to 330 nm were achieved. A greater concentration and longer deposition time could result in thicker CNT layers ranging from several tens to hundreds of nanometers. In this work, 1.5 wt% CNT was utilized with 20 depositions, producing a CNT approximately 200 nm thick.

Various shapes of patterns were also achieved to show the versatility of directly patterning CNT on elastomer substrate. Rectangular and circular shapes, with a width and diameter of 500 μm, respectively, were fabricated as shown in Figure 3D,E. Rectangular arrays with a width of 500 μm, pitch of 500 μm, and height of 1 mm was also achieved using direct patterning with an elastomer substrate of 5:1 Ecoflex and PDMS, as shown in Figure 3F, with the selected “OPP 30” mask and 30 s of O_2_ plasma treatment.

### 3.4. CNT Pattern in a Sensing Platform

The electrical and mechanical properties of CNT patterned on an elastomer substrate demonstrate the feasibility of applying our direct patterning method as a sensing platform. To measure the electrical and mechanical properties of the CNT patterned sensor as it stretches, the CNT pattern layer encapsulated with an elastomer or electrodes before the strain was applied. The used method was the same as that previously introduced in Section 2.5. 

**Figure 4:**
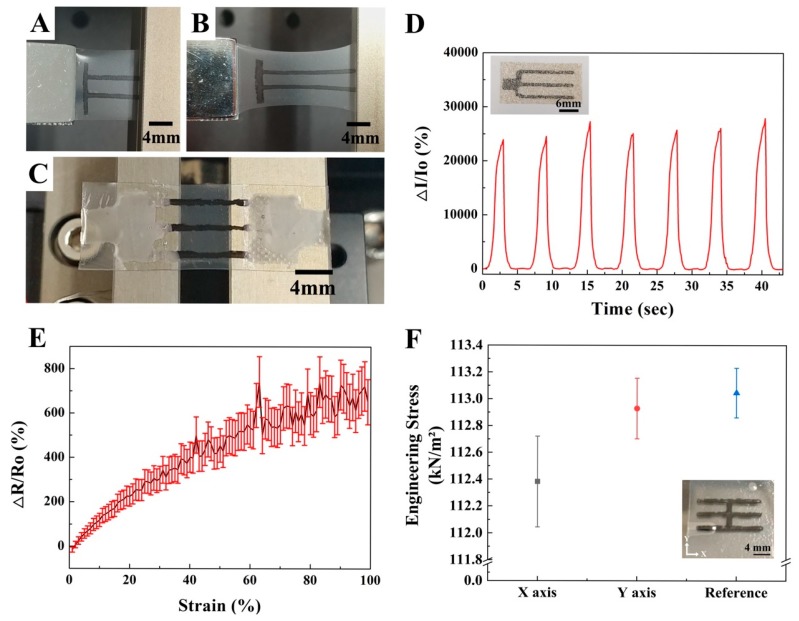
Electrical and mechanical properties of the encapsulated CNT double layer sensor. Images of the ‘π’ pattern when (**A**) 0% and (**B**) 100% strain were applied. (**C**) Double layer sensor with an Ag nanowire electrode on the CNT. (**D**) Time–current curves of CNT masked by polyimide film and measured by repeatedly stretching samples at 100% strain. (**E**) Changes in resistance when the sensor is stretched to 100%. (**F**) The average values and standard deviation of engineering stress of three CNT line patterns along the X-axis, one along the Y-axis and bare elastomer substrate (mixed by Ecoflex:PDMS = 5:1).

Before the electrical signal measurements were taken, it was necessary to ensure that the CNT pattern remained intact while encapsulated in the elastomers. To confirm that, the CNT patterns were repeatably stretched while 100% strain was applied. The repetitive deformation test was resulted in CNT forming a “π” pattern, with two line-shaped CNT patterns deposited in the X-axis and one on the Y-axis, as shown in Figure 4A (0% strain) and Figure 4B (100% strain). One side of the sample was connected to the digital push/pull gauge and the other was fixed to the motorized stage for the repeated extension. Even after dozens of repeated stretching tests, the pattern kept its shape and the elastomer was not damaged.

Two kinds of materials were utilized as electrode materials: An Ag nanowire ink and the Ag flake-Ecoflex mixture introduced in Section 2.5. The Ag electrode layers work as a protective layer and as an external electrode connected to probe station during the measurements of the electrical signals. By depositing Ag nanowire ink with MDD, electrodes are placed at both ends of the CNT pattern, as shown in Figure 4C. To connect the electric wire with Ag nanowire electrodes, part of upper layer of the elastomer needed to be stripped after the elastomer was spin coated on the CNT patterns. As the exposed Ag nanowire is not suitable for the repeated strain test, we used an Ag-Ecoflex elastomer as an upper layer to create a sensor with an encapsulated CNT pattern. The Ag flake and Ecoflex mixture was spin-coated on the CNT pattern as the deposited conductive layer, as seen in the inset image of Figure 4D. 

To obtain a change in resistance while the strain was applied, one part of the sensor was fixed to a stationary table and the other to a motorized stage. When the latter deformed the sensor at a speed of 2 mm/s, the change in the ratio of resistance to the original resistance ($\frac{\Delta R}{R0}\;\left(\%\right))$ shifted the incremental curve from 0% to about 770% as the strain increased from 0% to 100%, as shown in Figure 4E. While the change in the ratio of resistance to the original resistance was being measured, a 5 V charge was supplied to the sensor. The resistance gradually increased according to the applying strain, up to 100%; a strain greater than 100% was not tested.

The Ag-Ecoflex layer that encapsulated the CNT patterns was utilized to measure the electrical properties resulting from applying a strain repeatedly over time. Strains of 0% to 100% were applied repeatedly to the elastomer and a current of 5 V was also applied. The Ag flakes and Ecoflex were mixed at a 4:5 ratio and cured at 90 °C for an hour after spin coating. The sensor was prepared with a width of 1.8 cm and a height of 1.2 cm. The CNT patterns were deposited in the same manner as the sensors were, as shown in Figure 4C. The CNT pattern on the elastomer layer was encapsulated with an Ag-Ecoflex layer that has a great recovery from repetitive strain.

The currents were measured under the same conditions as the resistance measurements were taken, as shown in Figure 4E. We obtained a constant cyclicity in the change rates of the currents during the measurements, as shown in Figure 4D. The current increased about 2000 times as the sensors were stretched and cycled back to their original values when the strain was reduced. This repetitive pattern resulted in the CNT pattern deposited by MDD and can be used to fabricate a reliable and stretchable device. As shown in Figure 4F, axial stress values were measured to show the change in mechanical properties caused by the CNT patterns. These were formed in three lines along the X-axis and one line along the Y-axis, as shown in the inset image of Figure 4F.

The CNT pattern that contained the elastomer double layer represented less engineering stress (kN/m²) compared to the reference double layer elastomer that did not include a CNT pattern. Engineering stress is calculated by dividing the average peak value of engineering stress by the initial cross section area of the stretched direction. The average of peak values was obtained when samples were stretched to 100% strain for 3 s in each axial direction five times. The double elastomer layer without the CNT pattern, which was the reference, had the highest engineering stress with a value of approximately 113.05 ± 0.19 kN/m². Otherwise, the strain along the X-axis and Y-axis of the strain pattern was approximately 112.38 ± 0.34 kN/m² and 112.93 ± 0.23 kN/m², respectively, as shown in Figure 4F. From the measured values, stress of CNT could be derived with a deduction of stress from the CNT pattern contained substrate and reference. The derived values were 0.67 ± 0.15 kN/m² and 0.12 ± 0.04 kN/m² along the X-axis and Y-axis of the strain, respectively. The values were obviously different according to the axis of strain; stress per unit length of CNT ($S_{unit}$) was also calculated in Figure S5 of supplementary material. From that, the CNT patterning method could be considered to be applicated in the tensile sensor for measuring the stress of multi-axis.

## 4. Conclusions

O_2_ plasma treatment was applied to a CNT pattern in several hundreds of nanometers thickness and placed on an elastomer substrate, and optimized to obtain a hydrophilic elastomer substrate. Ecoflex and PDMS mixed at a 5:1 ratio was utilized as an elastomer substrate. To optimize the O_2_ plasma treatment condition, the interfacial effect of substrate was analyzed using counting cross points of crack and contact angle measurement. For a stretchable sensor, mechanical and electrical properties are very important; therefore, we also measured the variation of a current based on the O_2_ plasma treatment and the strain–stress to validate the appropriate mechanical property of the substrate. For the direct patterning of the CNT, a sacrificial mask with a defined uniformity and sharpness was selected. The analysis shows that we could confirm all conditions needed for the direct patterning of the CNT on a thin elastomer substrate with good electrical and mechanical properties. Finally, we created a CNT layer containing a double layer elastomer that could be applied as a sensor to measure mechanical properties under optimized fabrication conditions. Our direct patterning methods were very simple and could produce uniform, thin, sharp, and varied patterns on an elastomeric substrate. Therefore, we hope that our methods can be utilized widely in various fields in which electrodes based on stretchable substrate with good electrical and mechanical properties and a fabrication process that is not complicated and based on demonstrated patterns.

## Figures and Tables

**Table 1:** The number of the cross points in the crack lines and mean value and standard deviation for contact angles of water drop on various substrates, according to the treatment time of O_2_ plasma.

	Mixture Ratio	100:0		10:1		5:1	
Plasma Time (s)		Contact Angle (°)	Cross Points	Contact Angle (°)	Cross Points	Contact Angle (°)	Cross Points
0		102.32 ± 3.75	0	102.73 ± 1.29	0	96.51 ± 2.36	0
15		12.65 ± 2.42	0	9.76 ± 0.21	0	10.22 ± 0.46	0
30		10.65 ± 1.39	4.8 ± 0.36	11.05 ± 0.64	2.7 ± 1.15	10.14 ± 1.18	0.8 ± 0.94
45		11.02 ± 0.77	15.0 ± 5.72	11.54 ± 0.67	8.30 ± 1.46	11.51 ± 0.96	13.7 ± 2.71

**Table 2:** Thickness of each type of mask.

Name	Materials	Thickness (μm)
OHP 100	Overhead projector film (OHP)	100
OPP 30	Oriented polypropylene (OPP)	30
PI 25	Polyimide (PI)	25
Teflon 25	Teflon	25
Teflon 50	Teflon	50

## Supplementary Materials

The following are available online at https://www.mdpi.com/2072-666X/10/8/530/s1: Figure S1: Detailed fabrication processes for the CNT-based sensor with meniscus-dragging deposition (MDD), Figure S2: Cross point analysis for quantitative analysis of the crack, Figure S3: Elastomer cracks and contact angles for specific conditions, Figure S4: Cross sectional image of double elastomer layer, Figure S5: Comparison of $S_{unit}$ along the X and Y-axis, Table S1: Numeric data for contact angles from the O_2_ plasma treatment time, Table S2: Numeric data for contact angles after 15 min and 30 min, Table S3: Numeric data for cross points of the crack lines depending on O_2_ plasma treatment time and substrate ration, Table S4: Thickness of double elastomer layer.
