# Supplementary material for: Direct Patterning of a Carbon Nanotube Thin Layer on a Stretchable Substrate
Source: Micromachines (Basel). 2019 Aug 11;10(8):530. doi: 10.3390/mi10080530 (PMC6722655; doi:10.3390/mi10080530)
Supplement: Supplementary file 1 [file micromachines-10-00530-s001.pdf]

Supplementary Materials: Direct Patterning of a Carbon Nanotube Thin Layer on a Stretchable Substrate

Eunji Lee, Hye Jin Kim, Yejin Park, Seungjun Lee, Sae Youn Lee, Taewon Ha, Hyun-joon Shin, Youngbaek Kim and Jinsik Kim

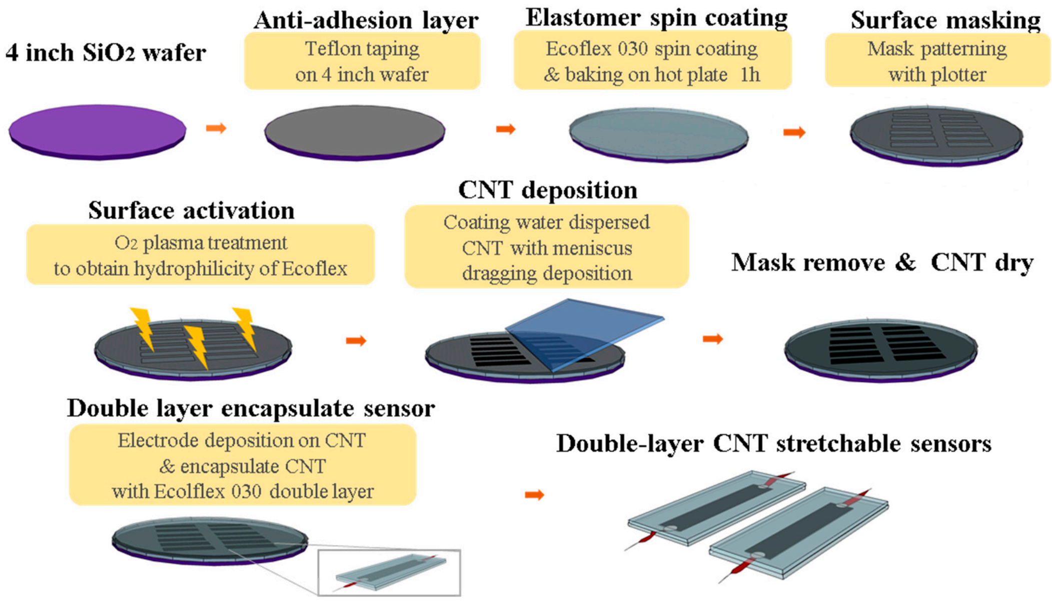

Figure S1. Fabrication method of patterning a double-layered carbon nanotube (CNT) sensor with meniscus dragging deposition (MDD).

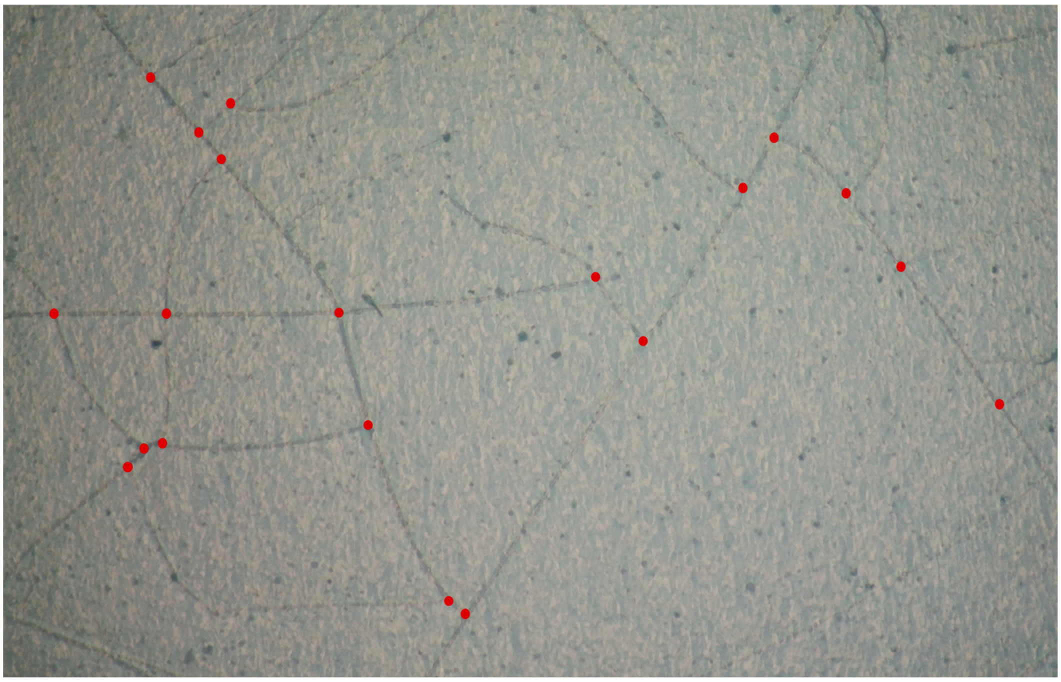

Figure S2. Cross points of crack lines that appeared after O_2 plasma treatment for 45 s on Ecoflex:PDMS = 5:1 substrates.

Table S1. Contact angles between water drop and substrate varied according to the O_2 plasma treatment time (unit: Degree).

O_2 Plasma Treatment Time (s)	Ecoflex 100% Contact Angle	Ecoflex:PDMS = 10:1 Contact Angle	Ecoflex:PDMS = 5:1 Contact Angle
0	102.32 ± 3.75	102.73 ± 1.29	96.51 ± 2.36
15	12.65 ± 2.42	9.76 ± 0.214	11.07 ± 0.57
30	10.65 ± 1.39	11.05 ± 0.64	11.28 ± 0.33
45	11.02 ± 0.77	11.54 ± 0.67	12.36 ± 1.07
60	11.30 ± 0.11	10.51 ± 0.32	11.98 ± 0.97
90	13.47 ± 2.24	12.73 ± 1.77	14.26 ± 1.70

Table S2. Contact angles estimated 15 min and 30 min after plasma treatment on the 5:1 ratio substrate (unit: Degree).

O_2 Plasma Treatment Time (s)	Contact Angle after 15 min	Contact Angle after 30 min
0	96.51 ± 2.36	96.51 ± 2.36
15	11.07 ± 0.57	16.17 ± 3.22
30	11.28 ± 0.33	18.95 ± 4.14
45	12.36 ± 1.07	30.47 ± 3.48
60	11.98 ± 0.97	22.22 ± 3.86
90	14.26 ± 1.70	21.80 ± 3.05

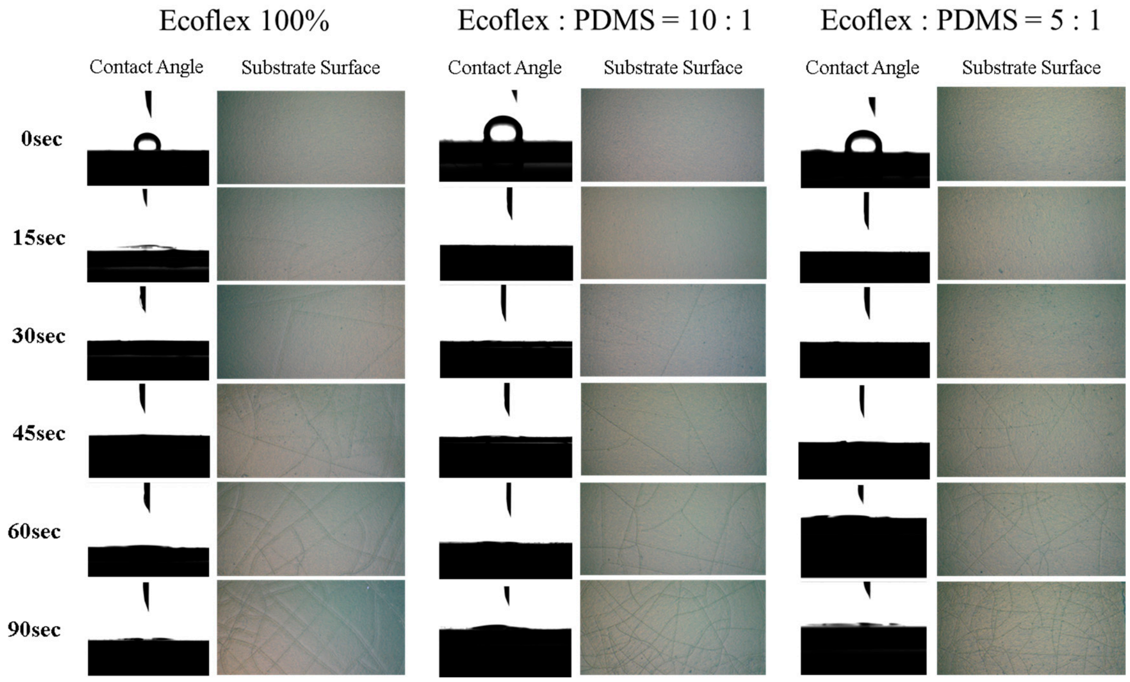

Figure S3. Optical images of contact angles and surface cracks of elastomer. Contact angle and intensity of surface cracks vary according to the plasma treatment time and substrate mixture ratio; Ecoflex 100%, Ecoflex:PDMS = 10:1, and Ecoflex:PDMS = 5:1.

Table S3. Variation in cross points of crack lines according to treatment time and substrate mixture ratio.

O_2 Plasma Treatment Time (s)	Ecoflex 100%	Ecoflex:PDMS = 10:1	Ecoflex:PDMS = 5:1
0	0	0	0
15	0	0	0
30	4.8 ± 0.36	2.7 ± 1.15	0.8 ± 0.94
45	15.0 ± 5.72	8.3 ± 1.46	13.7 ± 2.71
60	23.0 ± 2.88	23.8 ± 3.04	67.2 ± 15.53
90	36.5 ± 14.81	128 ± 14.97	125.5 ± 2.73

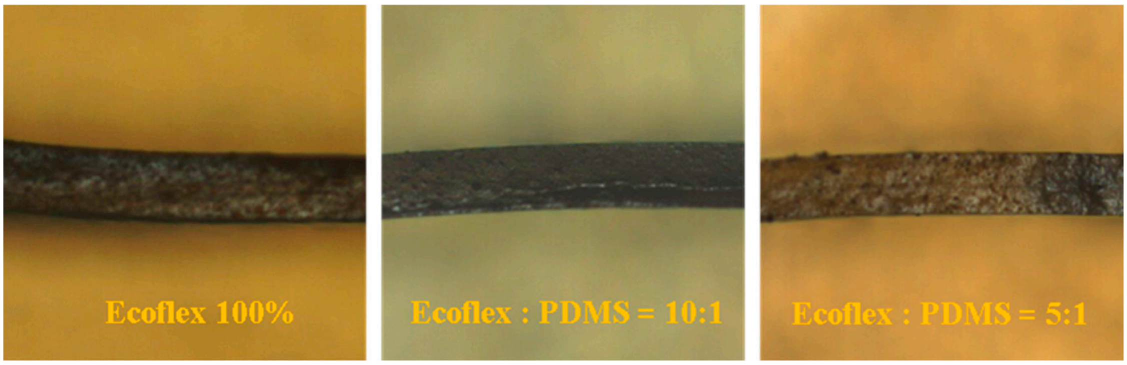

Figure S4. Cross-sectional images of the double elastomer layer according to the mixture ratio of Ecoflex and polydimethylsiloxane (PDMS).

Table S4. Thickness of the double elastomer layer according to the mixture ratio of Ecoflex and polydimethylsiloxane (PDMS).

Ecoflex:PDMS Ratio	Thickness (μm)
Ecoflex 100%	173.41 ± 1.91
10:1	164.15 ± 1.14
5:1	163.57 ± 1.79

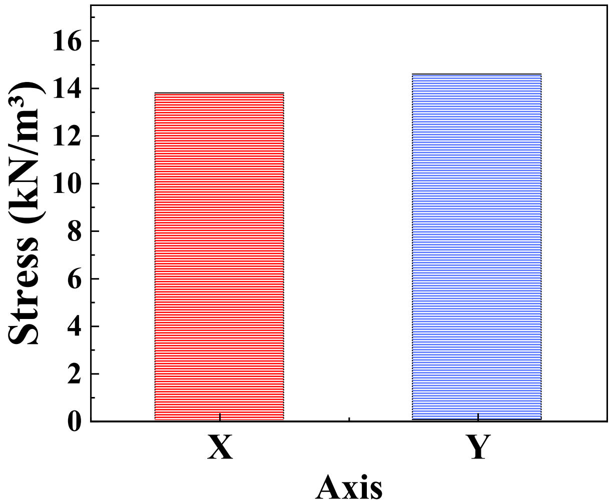

Figure S5. S_{unit} of the CNT pattern along the axis calculated using Equation (3). S_{unit} of CNT pattern along the X-axis is approximately 13.8 kN/m^3 and that along the Y-axis is approximately 14.6 kN/m^3.

The mechanical test of the double layer sensor, the engineering stress of the reference, and the X- and Y-axis of the sensor had different values, as shown in the manuscript, Figure 4F. As the

pattern of the CNT was different according to the two axes, we analyzed the correlation between the difference in the stress value of axis and reference to figure out whether the CNT pattern was affecting the X- and Y-axes in the same manner. To analyze the effect of the CNT pattern on the mechanical properties of the sensor, the difference in axial stress can be analyzed with the following Equation (S1):

$$S_{unit} = \frac{\sigma_R - \sigma_{axis}}{L_{axis}}, \quad \text{(S1)}$$

where S_{unit} indicates the stress per unit length of the CNT pattern, σ_R is the engineering stress of the reference (the bare elastomer), σ_{axis} is the engineering stress of the X- or Y-axes, and L_{axis} is the length of the CNT pattern along the stretched direction. The equation can derive only the mechanical stress of the CNT pattern by subtracting the axial stress from the stress of the bare substrate. The values are divided by the length of the deposited patterns along the stretched direction and measured engineering stress of the X- or Y-axes in Figure 4F were also utilized; the strain along the X-axis and Y-axis was approximately 112.38 ± 0.34 kN/m^2 and 112.93 ± 0.23 kN/m^2, respectively. L_{x} was 48 mm, the total length of three lines patterns on the x-axis and L_{y} was 8 mm, the length of the pattern deposited along the Y-axis. The values of S_{unit} were compared to confirm the uniformity of the CNT pattern; the more uniform the CNT pattern deposited is, the closer in value the S_{unit} of both axes should be. According to Equation (S1), the S_{unit} of the CNT pattern along the X-axis was approximately 13.8 kN/m^3, while it was approximately 14.6 kN/m^3 along the Y-axis, as shown in supplementary Figure S5. The difference in the values of S_{unit} along the X- and Y-axes was approximately 0.8 kN/m^3. By analyzing the S_{unit} of the CNT pattern, we found that CNT patterns lowered the stress value of substrate by as much as their pattern length. The CNT patterns tested were also well-designed and differed in number or length based on the axis; therefore, the sensor had different mechanical properties based on the axis. Furthermore, we considered that the CNT patterning demonstrated a fair uniformity based on the similar values of the stress per unit length of the CNT pattern in which there was no stress from the substrate.

© 2019 by the authors. Submitted for possible open access publication under the terms and conditions of the Creative Commons Attribution (CC BY) license (http://creativecommons.org/licenses/by/4.0/).
